# Prediction of relapsed/refractory primary central nervous system lymphoma using pre-chemotherapy ITSS grade on SWI and pre-/post-chemotherapy ADC parameters on DWI

**DOI:** 10.1186/s40644-026-01042-8

**Published:** 2026-04-30

**Authors:** Yijing Zhao, Feiman Yang, Meina Li, Feifei Yu, Na Lin, Dairong Cao, Zhen Xing

**Affiliations:** 1 Department of Radiology, The First Affiliated Hospital, Fujian Medical University, Fuzhou, 350005 China; 2 Department of Radiology, National Regional Medical Center, Binhai Campus of the First Affiliated Hospital, Fujian Medical University, Fuzhou, 350212 China; 3 Fujian Key Laboratory of Precision Medicine for Cancer, The First Affiliated Hospital, Fujian Medical University, Fuzhou, 350005 China; 4 Key Laboratory of Radiation Biology of Fujian Higher Education Institutions, The First Affiliated Hospital, Fujian Medical University, Fuzhou, 350005 China

**Keywords:** Primary central nervous system lymphoma, Relapsed/refractory, Susceptibility weighted imaging, Diffusion weighted imaging

## Abstract

**Objectives:**

The purpose of this study was to evaluate the role of SWI and DWI in predicting the relapsed/refractory primary central nervous system lymphoma (R/R PCNSL).

**Materials and methods:**

Seventy-seven patients with histologically confirmed PCNSL were enrolled. Patients were divided into R/R group (*n* = 40) and Non-R/R group (*n* = 37), according to follow-up outcomes. Demographics, pathological indicators, conventional MRI characteristics, pre-chemotherapy intratumoral susceptibility signal (ITSS) grade, ADC parameters, including pre-chemotherapy relative minimum ADC (rADC_min−pre_) and relative mean ADC (rADC_mean−pre_), post-chemotherapy relative minimum ADC (rADC_min−post_) and relative mean ADC (rADC_mean−post_), as well as the change in relative minimum ADC (rADC_min−change_) and relative mean ADC (rADC_mean−change_), were compared between the two groups. Receiver operating characteristic curves and logistic regression analysis were used to assess the predictive performance.

**Results:**

Compared with Non-R/R PCNSL group, R/R PCNSL group showed significantly lower Ki-67 index (*P* = 0.004) but higher pre-chemotherapy ITSS grade (*P* < 0.001). The comparison of rADC_min−pre_ and rADC_mean−pre_ between the two groups showed no significant differences (*P* > 0.05). However, among the 50 patients with available post-treatment ADC data, rADC_min−post_, rADC_mean−post_, rADC_min−change_ and rADC_mean−change_ in R/R group were significantly lower than in Non-R/R group (all *P* < 0.001). The predictive performance of rADC_min−post_, rADC_mean−post_, rADC_min−change_ and rADC_mean−change_ was comparable to that of pre-chemotherapy ITSS grade for R/R PCNSL (0.894 vs. 0.714, 0.867 vs. 0.714, 0.877 vs. 0.714, 0.814 vs. 0.714, all *P* > 0.05), but superior to that of Ki-67 index (0.894 vs. 0.665, 0.867 vs. 0.665, 0.877 vs. 0.665, 0.814 vs. 0.665, all *P* < 0.05).

**Conclusions:**

The pre-chemotherapy ITSS grade, rADC_min−post_, rADC_mean−post_, rADC_min−change_ and rADC_mean−change_ may serve as preferable imaging biomarkers for predicting R/R PCNSL, and compare favorably with Ki-67 index.

**Supplementary Information:**

The online version contains supplementary material available at 10.1186/s40644-026-01042-8.

## Introduction

Primary central nervous system lymphoma (PCNSL) accounts for approximately 4% of all newly diagnosed malignant central nervous system tumors and 4–6% of all extranodal lymphomas [1]. Among PCNSL cases, diffuse large B-cell lymphoma (DLBCL) is the most common histopathological type, comprising more than 90% of cases [2]. High-dose methotrexate (HD-MTX)-based combination chemotherapy has been widely adopted as the standard treatment for newly diagnosed PCNSL patients [3]. However, despite the chemosensitivity of PCNSL, 15–50% of patients are unresponsive to first-line therapy or experience relapse after an initial response [4–6]. Such patients are classified as relapsed/refractory (R/R) PCNSL, a grouping widely used in previous studies [7–9]. Although R/R PCNSL covers a broad spectrum of biologically distinct subgroups, including primary refractory, early relapse, and late multiple relapse, all share poor responses to conventional therapy and similar unfavorable prognosis, highlighting the need for more proactive approaches such as molecular targeted therapy, immunotherapy, autologous stem cell transplantation, and whole brain radiotherapy to improve clinical outcomes.

Currently, the prediction of R/R PCNSL relies on clinical indicators such as age, Karnofsky Performance Status, involvement of deep brain structures, cerebrospinal fluid protein levels, serum lactate dehydrogenase levels, and molecular biomarkers [1, 10]. However, most existing indicators require invasive methods and remain controversial. Thus, there is an urgent need to explore a non-invasive method to identify these patients early in their disease course. Conventional magnetic resonance imaging (cMRI) serves as a routine tool for assessing tumor morphological characteristics. Recently, a few studies have illustrated that baseline tumor size and lesion location are associated with overall survival and progression-free survival [11, 12]. In addition, Niparuck et al. [13] reported that multiple brain lesions were associated with favorable outcomes, yet other reports showed no significant difference [14]. Therefore, the value of cMRI features in predicting R/R PCNSL remains unclear and needs further validation.

Susceptibility-weighted imaging (SWI) has been increasingly used to reflect histological features of intracranial tumors by quantifying the intra-tumoral susceptibility signal (ITSS), which is typically invisible on cMRI [15, 16]. It has been reported that SWI may have potential in distinguishing different genomic subtypes of PCNSL [17]. However, studies exploring the role of SWI in predicting R/R PCNSL are extremely limited. Although Deguchi et al. [16] reported that the presence of ITSS before chemotherapy was significantly associated with the efficacy of HD-MTX treatment, its clinical utility requires validation through prospective large-sample studies due to the small sample size of this study. Furthermore, highly aggressive PCNSL induces immature tumor vasculature [18], resulting in inadequate perfusion, hemorrhage, and thus higher ITSS grade, which may mechanistically underlie a poor chemotherapeutic response. Diffusion-weighted imaging (DWI) is a non-invasive technique that reflects tumor cellularity together with the extracellular space by measuring the apparent diffusion coefficient (ADC) value. The relationship between pre-chemotherapy ADC values and treatment response or prognosis of PCNSL has only been reported in small-sample studies with inconsistent results [14, 19]. Moreover, previous studies have demonstrated that chemotherapy reduces cell density primarily through apoptosis and/or cell death, leading to increased water molecule diffusion and higher ADC values [20, 21]. Accordingly, post-chemotherapy ADC values may help identify patients at high risk for tumor refractoriness to HD-MTX-based treatment. Thus, the predictive value of pre-chemotherapy ADC values remains inconclusive, and post-chemotherapy ADC values may serve as imaging biomarkers for predicting R/R PCNSL.

In summary, reliable non-invasive biomarkers for prediction of R/R PCNSL remain scarce, and the predictive performance of SWI and DWI in this context has not been thoroughly examined. Thus, this study aims to investigate the potential of SWI and DWI in predicting R/R PCNSL, providing reliable imaging biomarkers for clinical risk stratification and personalized treatment strategies.

## Materials and methods

### Patients

**Fig. 1:**
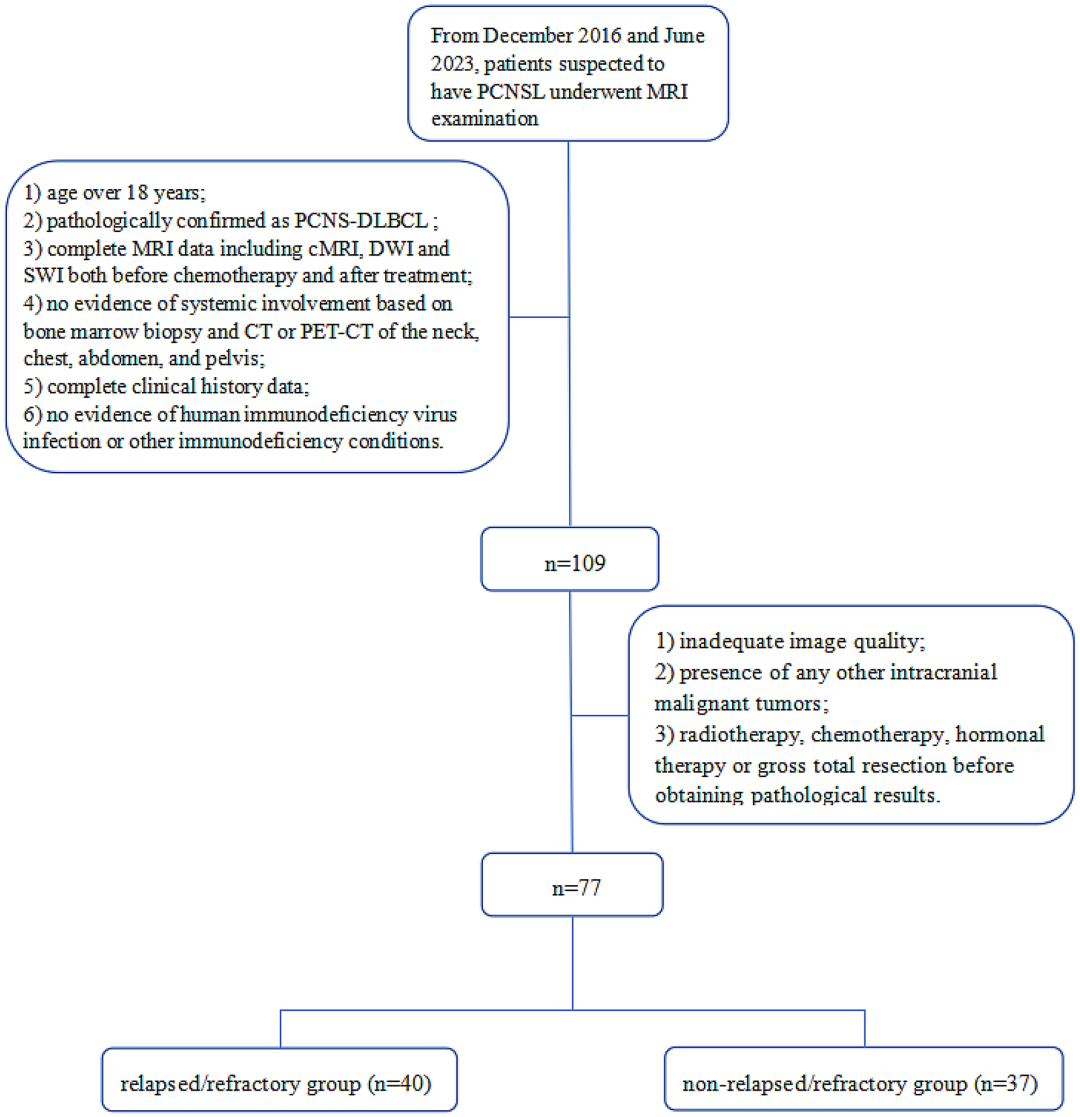
The workflow for patient selection

This study was approved by the ethics committee of our hospital, and informed consent was waived from all patients. The workflow for patient selection is shown in Fig. 1. A total of 109 patients with pathologically confirmed PCNSL at our institution between December 2016 and June 2023 were consecutively enrolled according to the following inclusion criteria: (1) age over 18 years; (2) pathologically confirmed as primary central nervous system diffuse large B-cell lymphoma (PCNS-DLBCL) based on the 2021 fifth edition of the WHO CNS 5 classification criteria [22]; (3) complete MRI data including cMRI, DWI and SWI both before chemotherapy and after treatment; (4) no evidence of systemic involvement based on bone marrow biopsy and computed tomography (CT) or positron emission tomography/computed tomography (PET-CT) of the neck, chest, abdomen, and pelvis; (5) complete clinical history data; (6) no evidence of human immunodeficiency virus infection or other immunodeficiency conditions. The exclusion criteria were as follows: (1) inadequate image quality; (2) presence of any other intracranial malignant tumors; (3) radiotherapy, chemotherapy, hormonal therapy or gross total resection before obtaining pathological results. Finally, 77 patients were included in this study.

### Therapeutic Evaluation and Follow-up

All patients received HD-MTX-based combination chemotherapy (MTX dose ≥ 3.0 g/m²) for 4–6 cycles, and other chemotherapeutic agents included temozolomide, rituximab and lenalidomide. Chemotherapy response was assessed based on the 2005 International PCNSL Collaborative Group (IPCG) response assessment criteria [23]: (1) complete response (CR): All enhancing lesions disappear on contrast-enhanced MRI; (2) partial response (PR): The volume of enhancing lesions decreases by ≥ 50% on contrast-enhanced MRI. (3) progressive disease (PD): The volume of enhancing lesions increases by ≥ 25% on contrast-enhanced MRI or the appearance of new enhancing lesions. (4) stable disease (SD): Lesions show no significant change on contrast-enhanced MRI, and the absence of any new lesions (not meeting PR or PD criteria). Patients achieving CR or PR at the end of first-line chemotherapy were considered responsive to treatment, while those with PD or SD were considered non-responsive. Follow-up was conducted every 3 months during the first 2 years after first-line chemotherapy and every 6 months thereafter. The follow-up period ended in December 2024, with minimum, median, maximum follow-up duration of 3, 21 and 56 months, respectively.

In this study, relapsed PCNSL was defined as lymphoma recurrence after CR to initial chemotherapy. The diagnosis of refractory PCNSL can be made if any one of the following criteria is met: (1) tumor reduction < 50% or disease progression after 4 cycles of standardized chemotherapy; (2) relapse within 6 months after CR; (3) two or more relapses after CR; (4) relapse after hematopoietic stem cell transplantation.

### Pathological data collection

Immunohistochemical results, including CD10, BCL-2, BCL-6, MUM-1, MYC, and Ki-67, were collected from the pathology system. Lymphoma subtypes were diagnosed by pathologists based on the WHO CNS 5 classification criteria. Hans classification was used to categorize DLBCL into germinal center B-cell-like (GCB) and non-germinal center B-cell-like (non-GCB) subtypes based on CD10, BCL-6, and MUM-1 results. Overexpression was defined as BCL-2 ≥ 50%, BCL-6 ≥ 30%, and MYC ≥ 40%, while high Ki-67 proliferation index was defined as ≥ 90% .

### MRI acquisition

All examinations were performed on 3.0 T MRI scanner (Verio, Skyra, Prisma; Siemens, Erlangen, Germany) using an identical 20-channel head/neck coil. MRI performed after the first chemotherapy cycle was acquired at a fixed interval of 7–9 days following completion of the cycle. The cMRI protocols included the following sequences: T1-weighted imaging (T1WI) (TR/TE = 250 ms/2.5 ms), T2-weighted imaging (T2WI) (TR/TE = 4000 ms/96 ms), fluid-attenuated inversion recovery imaging (FLAIR) (TR/TE = 9000 ms/94 ms), and contrast-enhanced T1WI (CE-T1WI) (TR/TE = 250 ms/2.5 ms). An FOV of 220 mm×220 mm, matrix = 256 × 256, slice thickness of 5 mm and gap of 1 mm were applied in all images. CE-T1WI images were acquired after administration of 0.1–0.2 mmol/kg of gadobenate dimeglumine (GD-BOPTA, Bracco, Italy).

SWI was performed using a three-dimensional fast low-angle shot technique with the following parameters: TR/TE = 27 ms/20 ms, FOV = 220 mm×220 mm, matrix = 256 × 256, slice thickness = 2 mm, slice gap = 0.4 mm. DWI was acquired with single spin echo-echo planar imaging (SE-EPI) sequence with the following parameters: TR/TE = 8200 ms/102 ms, FOV = 220 mm×220 mm, matrix = 128 × 128, slice thickness = 5 mm, slice gap = 1 mm and two b values (b = 0 and 1000 s/mm^2^). ADC maps were automatically generated on the MR system.

### Image post-processing and analysis

All images were processed on Siemens Syngo via workstations (Siemens, Germany) using standard software packages and analyzed via the picture archiving and communication system (PACS). All cMRI images were independently analyzed by 2 neuroradiologists (3 and 8 years of experience, respectively), using a blinded method, and disagreements were resolved by another neuroradiologist (15 years of experience). Tumors were assessed for (1) tumor location (supratentorial/infratentorial, cortical/deep); (2) number (single or multiple); (3) tumor size (maximum diameter, measured in an oblique 3D plane); (4) necrosis (present/absent); (5) peritumoral edema (measured in an oblique 3D plane, maximal distance from tumor margin to edema margin, mild: ≤ 1 cm, moderate: 1 cm < maximum distance ≤ 2 cm, severe: > 2 cm); (6) enhancement pattern (homogeneous/heterogeneous); (7) enhancement margin (clear/blurred).

For semiquantitative analysis of SWI data, the number of intralesional microhemorrhage was calculated and graded for each tumor. Microhemorrhage could be different from calcification based on the combination of phase images. The grading standard in the ITSS scoring system was set as follows: grade 0 = no hemorrhage; grade 1 = 1 to 10 dot-like hemorrhages; grade 2 = 11 to 20 dot-like hemorrhages; and grade 3 = more than 20 dot-like hemorrhages.

For evaluation of DWI data, areas with hemorrhage, necrosis, cystic degeneration, and blood vessels were carefully excluded during ROI placement. Five non-overlapping ROIs (0.1–0.2 cm^2^) were directly drawn on the ADC maps in regions exhibiting the visually lowest ADC values within the solid components of the tumors, and the minimum ADC value among the five ROIs was chosen as the pre-chemotherapy minimum ADC value (ADC_min−pre_), as reported by Zhou et al. [24]. Considering tumor heterogeneity, the measurement method for the mean ADC value is as follows: ROIs on each slice were carefully outlined along the boundaries of tumor enhancement on the CE-T1WI, and the corresponding ROIs were registered to the ADC map to generate the ADC values. The mean ADC value across all slices was defined as the pre-chemotherapy mean ADC (ADC_mean−pre_), as reported by Yu et al. [25]. Additionally, five similarly sized ROIs (0.1–0.2 cm^2^) were placed on ADC maps within the contralateral normal-appearing white matter (NAWM) to calculate their mean ADC value. The relative ADC_min−pre_ and ADC_mean−pre_ (rADC_min−pre_ and rADC_mean−pre_) ratios of the tumors were calculated by dividing the ADC_min−pre_ and ADC_mean−pre_ of the tumors by the mean ADC value of the contralateral NAWM, as reported by Ozturk et al. [17]. Similarly, the post-chemotherapy imaging was analyzed to obtain the relative minimum and mean ADC after the first chemotherapy cycle, designated as rADC_min−post_ and rADC_mean−post_, respectively. Finally, the changes in relative ADC after the first chemotherapy cycle were calculated as follows:


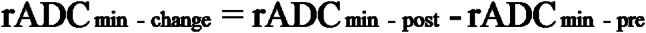



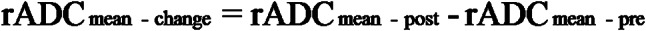
 An example of ADC measurements is illustrated in Supplemental Fig. 1

### Statistical analysis

Statistical analysis was performed using IBM SPSS Statistics 27.0, MedCalc 19.0.4, Graphpad Prism 8.0 and the R version 4.2.2 (http://www.Rproject.org). The Mann-Whitney *U* test was employed for comparison of ITSS grades between the Non-R/R group and R/R group. The Chi-square test was used for categorical data, with Fisher’s exact test applied in cases where any expected count in the contingency table was less than 5. Quantitative data were assessed for normality using the Kolmogorov-Smirnov test. All measurement data, including maximum diameter, rADC_min−pre,_ rADC_mean−pre_, rADC_min−post_, rADC_mean−post_, rADC_min−change_, and rADC_mean−change_, were normally distributed. All quantitative data were described as mean±standard deviation (SD). Two-sample *t* tests were used to compare the differences in the tumor maximum diameter, rADC_min−pre_, rADC_mean−pre_, rADC_min−post_, rADC_mean−post_, rADC_min−change_ and rADC_mean−change_. The diagnostic performance of univariate and multivariate parameters was evaluated using receiver operating characteristic (ROC) curve and binary logistic regression modeling, and then the area under the ROC curve (AUC), sensitivity, specificity, and Youden index were calculated. The different AUCs were compared using DeLong test, and Benjamini-Hochberg method was applied to adjust *P*-values for multiple comparisons. *P* < 0.05 was considered statistically significant. Inter-observer reliability was assessed using Cohen’s Kappa coefficient for categorical data and the intra-class correlation coefficient (ICC) for continuous data. Agreement levels were defined as follows: excellent (Kappa/ICC ≥ 0.75), good to fair (0.4 ≤ Kappa/ICC < 0.75), and poor (Kappa/ICC < 0.4).

## Results

A total of 77 patients were included in the study, and after the full course of first-line chemotherapy, 45 patients achieved CR, 14 patients had PR, 2 patients showed SD, and 16 patients experienced PD. This indicates that 18 patients did not respond to the HD-MTX-based combination chemotherapy (R/R vs. Non-R/R = 18 vs. 0), and 59 patients were responsive (R/R vs. Non-R/R = 22 vs. 37). By the end of the follow-up, 40 patients presented with R/R lesions. The cMRI features, ITSS grade, and rADC_min−pre_, rADC_mean−pre_, rADC_min−post_, rADC_mean−post_, rADC_min−change_, and rADC_mean−change_ demonstrated excellent inter-observer agreement, with all Kappa/ICC values exceeding 0.75.

### Demographic, pathological indicators and cMRI characteristics

The demographic and pathological indicators of the 77 patients were summarized in Table 1. The R/R group showed significantly lower Ki-67 index than that in Non-R/R group (*P* = 0.004), whereas there were no differences in terms of age, gender, and other pathological indicators between the two groups (all *P* > 0.05). The sensitivity, specificity, Youden index and AUC (95% confidence interval, 95% CI) of Ki-67 index for predicting R/R PCNSL were 0.600, 0.730, 0.330, and 0.665 (0.548, 0.768), respectively. The cMRI characteristics showed no statistically significant differences between the R/R group and Non-R/R group, including tumor location, number, tumor size, necrosis, peritumoral edema, enhancement pattern and enhancement margin (all *P* > 0.05) (Supplemental Table 1).

**Table 1:** Comparison of demographic, pathological indicators between the two groups

Gender	*R*/*R* group (*n* = 40)	Non-*R*/*R* group (n *=* 37)	*P* value
Male and Female	20/20	19/18	0.906
Age (mean ± SD)	59.95 ± 11.08	61.24 ± 10.10	0.595
Hans classification			0.212
non-GCB	25	28	
GCB	15	9	
Ki-67			**0.004** ^*^
<90%	24	10	
≥ 90%	16	27	
BCL-2			0.051
<50%	4	11	
≥ 50%	23	18	
negative	13	8	
BCL-6			0.734
<30%	5	6	
≥ 30%	21	20	
negative	14	11	
MYC			0.248
<40%	28	22	
≥ 40%	10	14	
negative	2	1	

Note: R/R, relapsed and refractory; SD, standard deviations; GCB, germinal center B-cell-like.*P < 0.05

### Evaluation of pre-chemotherapy ITSS grade

As shown in Table 2, the pre-chemotherapy ITSS grade was significantly higher in the R/R group compared to the Non-R/R group (*P* < 0.001). Representative cases are shown in Supplemental Figs. 1 and 2. The optimal threshold of pre-chemotherapy ITSS grade for predicting R/R PCNSL was 1, with corresponding sensitivity, specificity, Youden index and AUC (95% CI) of 0.275, 0.919, 0.194, and 0.714 (0.599, 0.811), respectively.

**Table 2:** Comparison of pre-chemotherapy ITSS grade between the two groups

	R/R group (*n* = 40)	Non-R/R group (*n* = 37)	*P* value
ITSS grade			< 0.001^*^
0	13	27	
1	16	7	
2	6	2	
3	5	1	

Note: ITSS, intratumoral susceptibility signal; R/R, relapsed and refractory. *P < 0.05

### Evaluation of ADC parameters

**Fig. 2:**
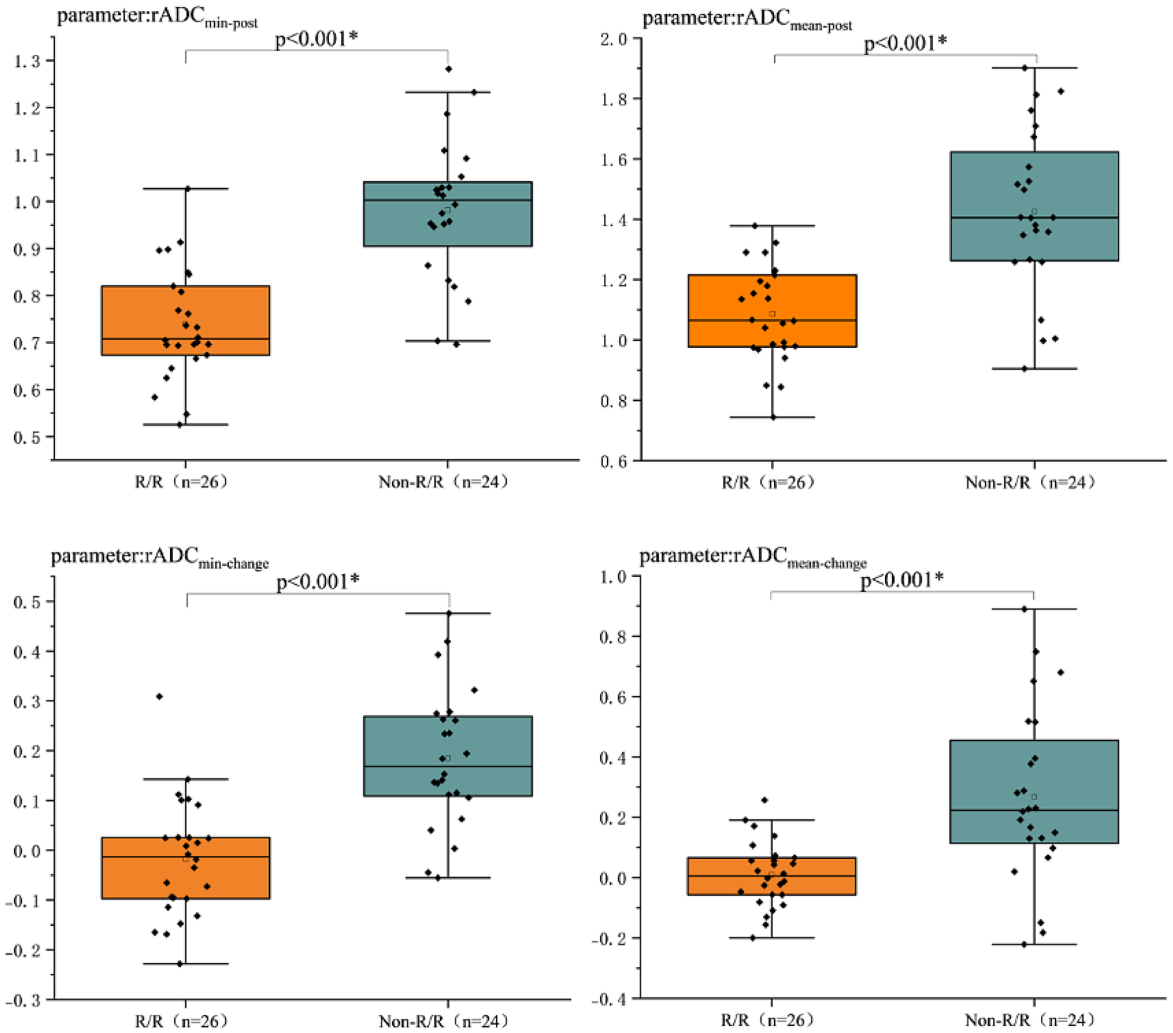
Box-plots of rADC_min−post_, rADC_mean−post_, rADC_min−change_ and rADC_mean−change_ between the two groups

**Fig. 3:**
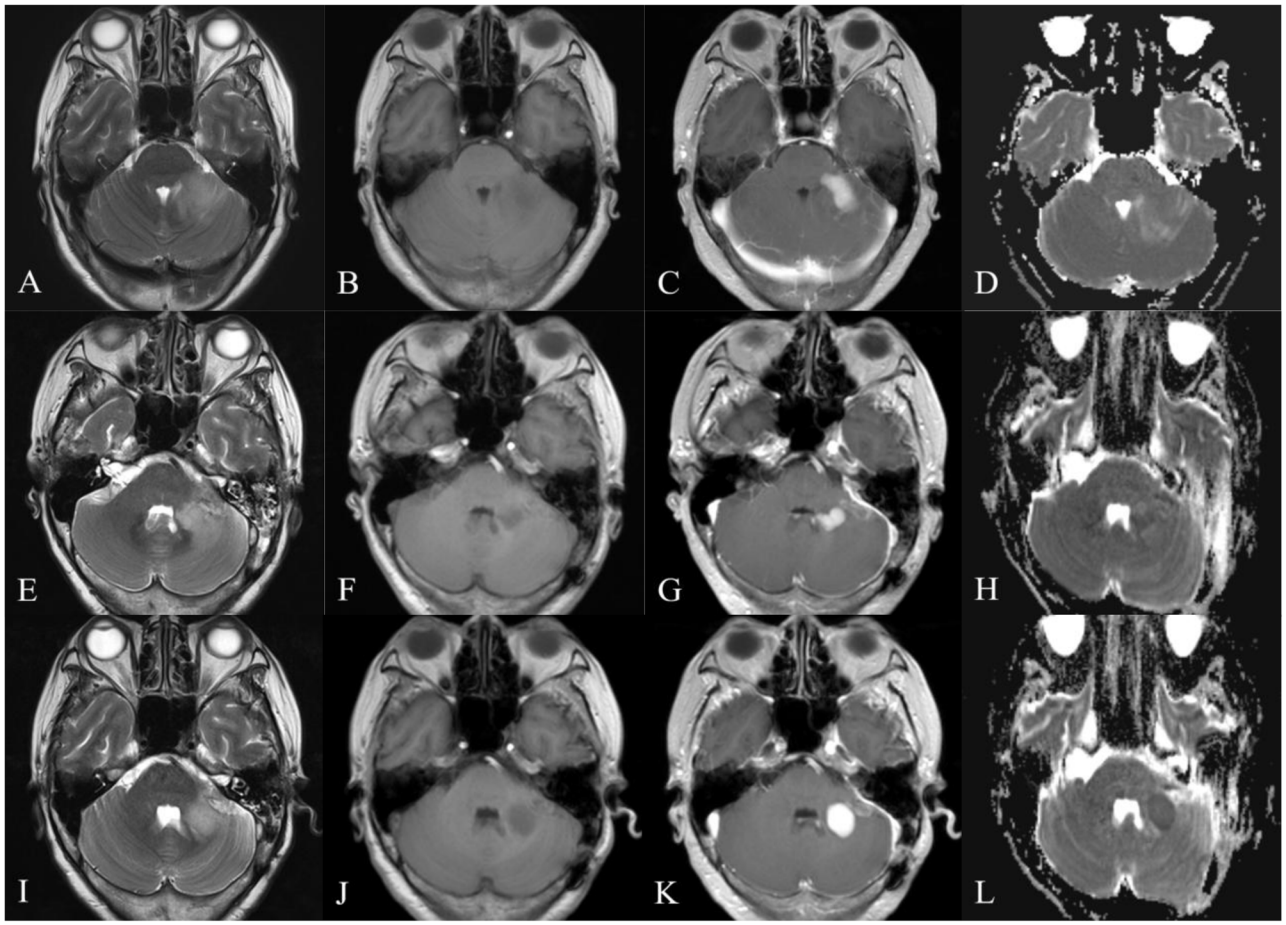
A 54-year-old female with relapsed/refractory PCNSL. Pretreatment MRI images (**A**-**D**). A solitary lesion was located in the left cerebellar peduncle, demonstrating mildly hyperintense signal on T2WI (**A**), hypointense signal on T1WI (**B**) and marked enhancement on CE-T1WI (**C**). The corresponding ADC map revealed rADC_min−pre_ of 0.885 and rADC_mean−pre_ of 1.159 (**D**). MRI images after a cycle of chemotherapy showed reduction in size compared to previous imaging (**E**-**G**). The corresponding ADC map demonstrated decreased signal intensity, with rADC_min−post_ of 0.747, rADC_mean−post_ of 1.012, rADC_min−change_ of -0.138, and rADC_mean−change_ of -0.147 (**H**). MRI images after the end of the whole chemotherapy exhibited interval enlargement in volume (**I**-**L**)

**Fig. 4:**
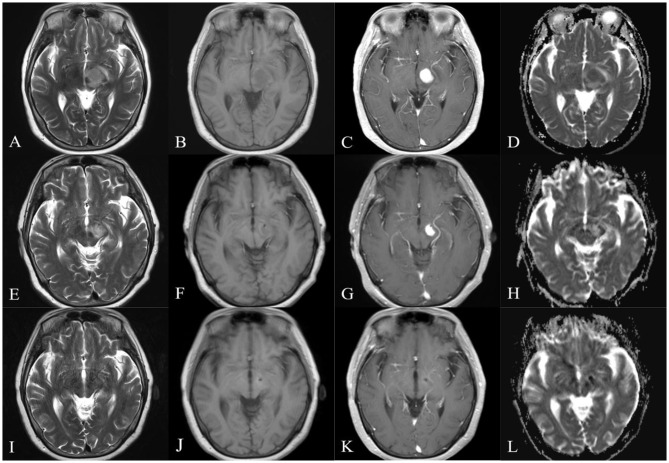
A 70-year-old female with non-relapsed/refractory PCNSL. Pretreatment MRI images (**A**-**D**). A solitary lesion was located in the left side of the midbrain, demonstrating mildly hyperintense signal on T2WI (**A**), hypointense signal on T1WI (**B**) and marked enhancement on CE-T1WI (**C**). The corresponding ADC map revealed rADC_min−pre_ of 0.892 and rADC_mean−pre_ of 1.021 (**D**). MRI images after a cycle of chemotherapy showed reduction in size compared to previous imaging (**E**-**G**). The corresponding ADC map demonstrated increased signal intensity, with rADC_min−post_ of 1.342, rADC_mean−post_ of 1.495, rADC_min−change_ of 0.450, and rADC_mean−change_ of 0.474 (**H**). MRI images after the end of the whole chemotherapy showed that the lesion had essentially disappeared (**I**-**L**)

**Fig. 5:**
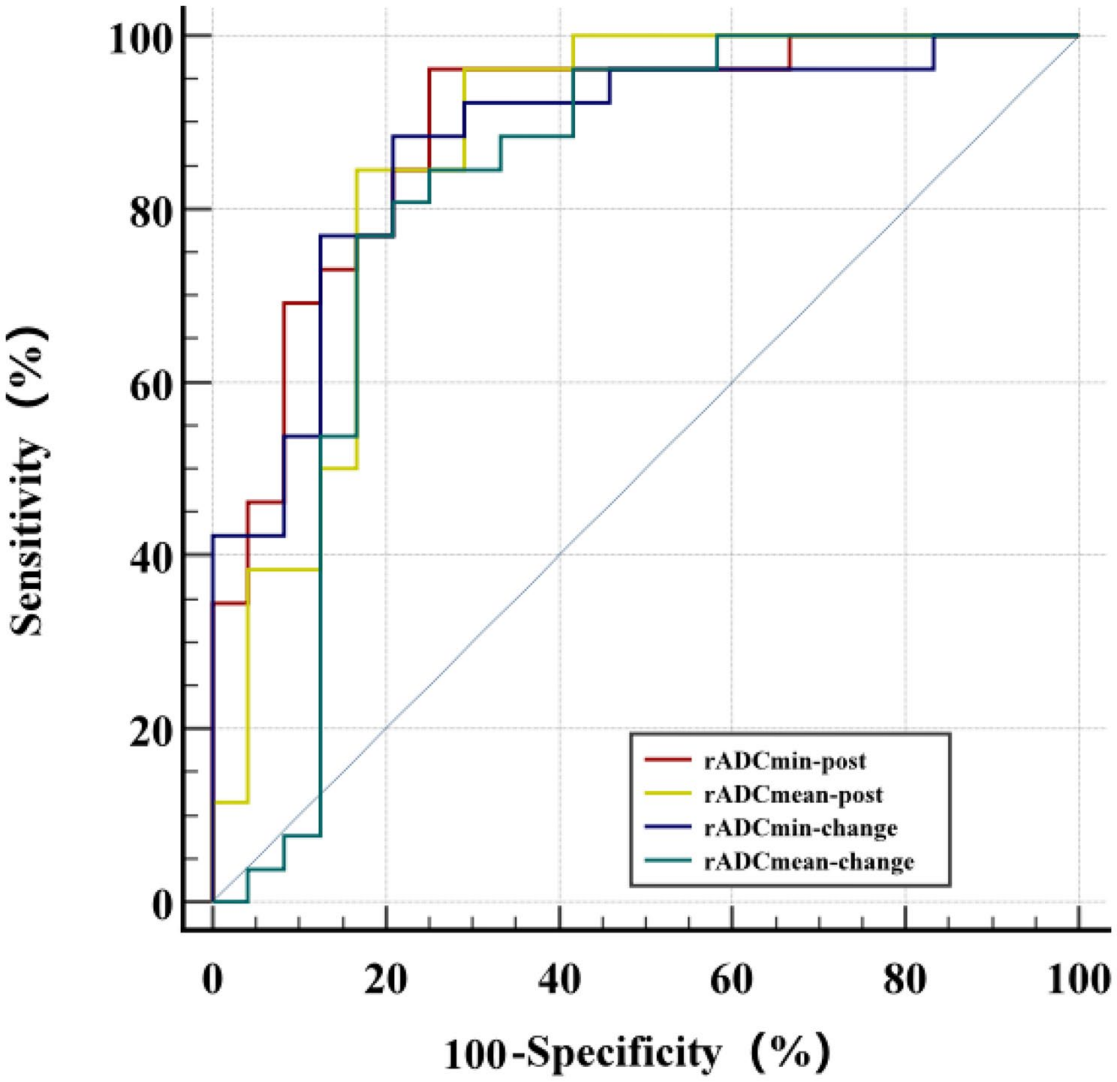
ROC curves of different models for predicting R/R PCNSL

The rADC_min−pre_ and rADC_mean−pre_ showed no significant differences between the R/R group and Non-R/R group (both *P* > 0.05) (Table 3). Owing to extensive hemorrhage or minimal residual enhancing tumor after the first chemotherapy cycle, which precluded accurate measurement, final ADC measurements were available for 50 PCNSL patients (R/R vs. Non-R/*R* = 26 vs. 24). No significant clinical or radiographic differences were observed between the excluded patients and those included in the ADC analysis (all *P* > 0.05). The rADC_min−post_, rADC_mean−post_, rADC_min−change_, and rADC_mean−change_ in the R/R group were significantly lower than those in the Non-R/R group (all *P* < 0.001) (Table 4; Fig. 2). Typical cases are shown in Figs. 3 and 4. Moreover, the predictive performance of the ADC parameters for R/R PCNSL is presented in Fig. 5. For the prediction of R/R PCNSL, the optimal thresholds with corresponding sensitivity, specificity, Youden index, and AUC (95% CI) were 0.913, 0.961, 0.750, 0.712, 0.894 (0.775, 0.963) for rADC_min−post_, 1.230, 0.846, 0.833, 0.680, 0.867 (0.741, 0.946) for rADC_mean−post_, 0.103, 0.885, 0.792, 0.676, 0.877 (0.755, 0.954) for rADC_min−change_, and 0.066, 0.769, 0.833, 0.603, 0.814 (0.679, 0.910) for rADC_mean−change_, respectively.

**Table 3:** Comparison of ADC parameters between the two groups before chemotherapy

	R/R group (*n* = 40)	Non-R/R group (*n* = 37)	*P* value
rADC_min−pre_	0.747 ± 0.103	0.781 ± 0.077	0.102
rADC_mean−pre_	1.081 ± 0.115	1.122 ± 0.158	0.198

Note: R/R, relapsed and refractory; rADC_min−pre_, relative minimum apparent diffusion coefficients before chemotherapy; rADC_mean−pre_, relative mean apparent diffusion coefficients before chemotherapy; date were expressed as means ± standard deviation

**Table 4:** Comparison of ADC parameters between the two groups after chemotherapy

	R/R group (*n* = 26)	Non-R/R group (*n* = 24)	*P* value
rADC_min−post_	0.739 ± 0.118	0.982 ± 0.148	< 0.001^*^
rADC_mean−post_	1.086 ± 0.160	1.426 ± 0.271	**< 0.001** ^*^
rADC_min−change_	-0.018 ± 0.118	0.185 ± 0.138	**< 0.001** ^*^
rADC_mean−change_	0.010 ± 0.109	0.267 ± 0.288	**< 0.001** ^*^

Note: R/R, relapsed and refractory; rADC_min−post_, relative minimum apparent diffusion coefficients after chemotherapy; rADC_mean−post_, relative mean apparent diffusion coefficients after chemotherapy; rADC_min−change_, the change in relative minimum apparent diffusion coefficients; rADC_mean−change_, the change in relative mean apparent diffusion coefficients; date were expressed as means ± standard deviation. *P < 0.05

### Comparison of predictive performance among different models

The comparison of predictive performance among different models for R/R PCNSL is shown in Table 5. The predictive performances of Ki-67 index and pre-chemotherapy ITSS grade were comparable for R/R PCNSL (0.665 vs. 0.714, *P* > 0.05), and their combination yielded no significant improvement (0.767 vs. 0.665, 0.767 vs. 0.714, *P* > 0.05). Moreover, the predictive performance of ADC parameters, including rADC_min−post_, rADC_mean−post_, rADC_min−change_, and rADC_mean−change_, was comparable to that of pre-chemotherapy ITSS grade for R/R PCNSL (0.894 vs. 0.714, 0.867 vs. 0.714, 0.877 vs. 0.714, 0.814 vs. 0.714, all *P* > 0.05), but superior to that of Ki-67 index (0.894 vs. 0.665, 0.867 vs. 0.665, 0.877 vs. 0.665, 0.814 vs. 0.665, all *P* < 0.05).

**Table 5:** The comparison of predictive performance among different models for predicting R/R PCNSL

Model	AUC (95%CI)	adjusted *P* value
Ki-67 vs. ITSS	0.665 (0.548, 0.768) vs. 0.714 (0.599, 0.811)	0.663
Ki-67 vs. Ki-67 + ITSS	0.665 (0.548, 0.768) vs. 0.767 (0.656, 0.855)	0.077
Ki-67 vs. rADC_min−post_	0.665 (0.548, 0.768) vs. 0.894 (0.775, 0.963)	**0.027** ^*^
Ki-67 vs. rADC_mean−post_	0.665 (0.548, 0.768) vs. 0.867 (0.741, 0.946)	**0.045** ^*^
Ki-67 vs. rADC_min−change_	0.665 (0.548, 0.768) vs. 0.877 (0.755, 0.954)	**0.043** ^*^
Ki-67 vs. rADC_mean−change_	0.665 (0.548, 0.768) vs. 0.814 (0.679, 0.910)	**0.045** ^*^
ITSS vs. Ki-67 + ITSS	0.714 (0.599, 0.811) vs. 0.767 (0.656, 0.855)	0.360
ITSS vs. rADC_min−post_	0.714 (0.599, 0.811) vs. 0.894 (0.775, 0.963)	0.055
ITSS vs. rADC_mean−post_	0.714 (0.599, 0.811) vs. 0.867 (0.741, 0.946)	0.122
ITSS vs. rADC_min−change_	0.714 (0.599, 0.811) vs. 0.877 (0.755, 0.954)	0.077
ITSS vs. rADC_mean−change_	0.714 (0.599, 0.811) vs. 0.814 (0.679, 0.910)	0.122
Ki-67 + ITSS vs. rADC_min−post_	0.767 (0.656, 0.855) vs. 0.894 (0.775, 0.963)	0.134
Ki-67 + ITSS vs. rADC_mean−post_	0.767 (0.656, 0.855) vs. 0.867 (0.741, 0.946)	0.324
Ki-67 + ITSS vs. rADC_min−change_	0.767 (0.656, 0.855) vs. 0.877 (0.755, 0.954)	0.234
Ki-67 + ITSS vs. rADC_mean−change_	0.767 (0.656, 0.855) vs. 0.814 (0.679, 0.910)	0.326
rADC_min−post_ vs. rADC_mean−post_	0.894 (0.775, 0.963) vs. 0.867 (0.741, 0.946)	0.607
rADC_min−post_ vs. rADC_min−change_	0.894 (0.775, 0.963) vs. 0.877 (0.755, 0.954)	0.747
rADC_min−post_ vs. rADC_mean−change_	0.894 (0.775, 0.963) vs. 0.814 (0.679, 0.910)	0.631
rADC_mean−post_ vs. rADC_min−change_	0.867 (0.741, 0.946) vs. 0.877 (0.755, 0.954)	0.864
rADC_mean−post_ vs. rADC_mean−change_	0.867 (0.741, 0.946) vs. 0.814 (0.679, 0.910)	0.911
rADC_min−change_ vs. rADC_mean−change_	0.877 (0.755, 0.954) vs. 0.814 (0.679, 0.910)	0.809

Note: AUC, area under the curve; CI, confidence interval; ITSS, intratumoral susceptibility signal; rADC_min−post_, relative minimum apparent diffusion coefficients after chemotherapy; rADC_mean−post_, relative mean apparent diffusion coefficients after chemotherapy; rADC_min−change_, the change in relative minimum apparent diffusion coefficients; rADC_mean−change_, the change in relative mean apparent diffusion coefficients. *adjusted *P* value < 0.05

## Discussion

In this study, we evaluated and compared pathological indicators, cMRI characteristics, pre-chemotherapy ITSS grades, and ADC parameters between R/R and Non-R/R PCNSL. Our results showed that R/R PCNSL was more likely to have lower Ki-67 index but higher pre-chemotherapy ITSS grade in comparison with Non-R/R PCNSL. Moreover, the rADC_min−post_, rADC_mean−post_, rADC_min−change_, and rADC_mean−change_ in the R/R group were significantly lower than those in the Non-R/R group. Additionally, the predictive performance of ADC parameters, including rADC_min−post_, rADC_mean−post_, rADC_min−change_, and rADC_mean−change_, was comparable to that of pre-chemotherapy ITSS grade for R/R PCNSL, but superior to that of Ki-67 index. Hence, SWI and DWI may provide valuable information for predicting R/R PCNSL.

Our study revealed no significant difference in gender and age between the R/R and Non-R/R groups, which was in satisfactory agreement with previous studies [26]. However, the Ki-67 index in the R/R group was lower than that in the Non-R/R group, which contradicts some previous studies. Studies reported by Cho et al. [27] and Liu et al. [28] suggested that high Ki-67 index might indicate poor prognosis in PCNSL patients. The discrepancy may be explained by differences in immunohistochemical testing conditions (e.g., staining protocols, antibody clones, and equipment) as well as the intratumoral heterogeneity of PCNSL. Furthermore, higher Ki-67 may correlate with increased chemosensitivity, and chemotherapy-mediated selection for low Ki-67, chemoresistant subpopulations might plausibly explain the lower Ki-67 level in R/R PCNSL. Additionally, this study found no significant difference in Hans classification, BCL-2, BCL-6, and MYC expression levels between the two groups. Therefore, pathological indicators alone have limited predictive value for identifying R/R PCNSL.

In our study, there was also no significant difference in cMRI features between the two groups, which was inconsistent with several previous findings. It has been reported that tumor size and infratentorial involvement are important variables related to PCNSL prognosis [11]. Furthermore, Niparuck et al. indicated that multiple brain involvements are associated with better prognosis [13]. The discrepant results could be attributed to our use of maximum tumor diameter for size assessment, unlike the volumetric measurement in previous studies, as well as the small sample sizes and highly subjective evaluation of cMRI features, which are prone to selection and measurement biases. Although cMRI features may provide information on treatment response and prognosis for PCNSL patients, their clinical utility may be limited by the absence of established quantitative indicators and the lack of consistent findings across published studies.

As an emerging MRI technique, SWI is distinguished by its superior sensitivity in the evaluation of tumor calcification, microbleeds, and angiogenesis. The ITSS grading system has been actively investigated for the differential diagnosis of PCNSL [29, 30]. However, only a limited number of studies focused on the relationship between the ITSS grade and the treatment and prognosis of PCNSL. In this study, R/R PCNSL exhibited a higher pre-chemotherapy ITSS grade in comparison with Non-R/R PCNSL. This finding was consistent with that of Deguchi et al. suggesting that pre-chemotherapy ITSS grade is significantly associated with the efficacy of HD-MTX treatment [16]. Previous studies have shown that the infiltration and destruction of brain parenchyma by advanced PCNSL rely on sufficient neovascularization, which accounts for the presence of neoangiogenesis and abundant microvascular density [31]. Furthermore, high CD105 expression, predominantly expressed in proliferating vessels, is associated with significantly shorter overall survival in PCNSL patients, and the characteristic perivascular growth pattern of tumor cells is also linked to a poorer prognosis [32, 33]. Therefore, we hypothesize that highly aggressive PCNSL promotes the formation of immature tumor vasculature, resulting in inadequate blood supply and thereby culminating in hemorrhage, which corresponds to a higher ITSS grade. Thus, our study suggests that the pre-chemotherapy ITSS grade may serve as a valuable indicator of aggressive behavior in PCNSL and may have potential clinical value in identifying high-risk R/R PCNSL patients. Additionally, it is worth emphasizing that SWI does not require the injection of contrast agents, and the ITSS grading system is relatively straightforward to assess, offering a simple and clinically practical imaging biomarker for predicting R/R PCNSL.

DWI is a functional MRI technique that can indirectly reflect tumor cellularity and extracellular space by ADC values. Evidence from the literature indicates that a lower ADC value was associated with a higher tumor proliferation index [34, 35]. Although several studies have investigated the association between DWI and chemotherapy response and prognosis in PCNSL in recent years, most are limited by small sample sizes, and the findings across these studies are inconsistent [14, 19, 36, 37]. In the current study, we found no significant differences in rADC_min−pre_ and rADC_mean−pre_ between the R/R and Non-R/R groups, and similar finding was also reported by Huang et al. [38]. However, Chien et al. [9] suggested that pre-chemotherapy ADC values may help predict R/R PCNSL. Heterogeneity in treatment regimens across studies may partly account for these discrepant results. In the study by Chien et al., some patients underwent surgical resection, which may be associated with a more favorable prognosis compared to biopsy alone [9, 39, 40].

Notably, this study showed that rADC_min−post_, rADC_min−change_, rADC_mean−post_, and rADC_mean−change_ were significantly lower in the R/R group than in the Non-R/R group. Currently, the evaluation of treatment efficacy in PCNSL patients primarily relies on assessing tumor volume changes before and after chemotherapy. However, volumetric changes are insensitive to early biological alterations induced by therapy and cannot monitor microstructural changes at the cellular level. Previous studies have confirmed that effective anticancer treatments increase water molecule diffusion [21]. Moreover, investigations in animal models have revealed that chemotherapy reduces cellularity via apoptosis and/or cell death, accompanied by histological changes such as an expansion of the extracellular space and increases in pleomorphic cells, giant cells, and apoptotic cells [20, 41]. These factors collectively contribute to a significant increase in ADC values in the short term. Conversely, the absence of an ADC increase after chemotherapy may indicate persistent hypercellularity within the lesion and an incomplete cytotoxic response to treatment, which could underlie the higher risk of R/R PCNSL. Furthermore, Huang et al. [38] reported that through dynamic changes of the ADC_min_ and diameter pre- and post-chemotherapy, the change in ADC_min_ value occurred in advance of changes in tumor diameter. In this study, we observed cases where lesion volume decreased after initial chemotherapy while ADC values paradoxically decreased, followed by increased tumor volume at the next follow-up. This finding further indicates that changes in ADC values may represent a more sensitive indicator of chemotherapy response and microstructural alterations than changes in tumor size. Therefore, the ADC parameters after the first chemotherapy cycle may serve as reliable imaging biomarkers for predicting R/R PCNSL. Additionally, we also conducted ROC curve and logistic regression analysis to explore the differential performance of Ki-67 index, pre-chemotherapy ITSS grades, and ADC parameters. The predictive performance of ADC parameters after the first chemotherapy cycle, including rADC_min−post_, rADC_mean−post_, rADC_min−change_, and rADC_mean−change_, was comparable to that of pre-chemotherapy ITSS grade for R/R PCNSL, but superior to that of Ki-67 index.

There are some limitations in our study. Firstly, this is a study with the retrospective, single-center design and relatively small patient population. Future studies with a prospective, multi-center design and a larger sample size are required to validate these findings. Secondly, we analyzed MR images from two different 3.0T scanners. However, we optimized the sequences to minimize the differences and normalized the ADC values to CNWM to diminish the potential bias of measurement. Thirdly, the post-treatment ADC parameter was only available in 50 patients. However, there was no significant difference in clinical or radiographic characteristics between patients included in and excluded from the ADC analysis, indicating that the missing data were random and did not introduce obvious selection bias into our study. Finally, ADC measurements were obtained via manually drawn ROIs. While this method is susceptible to operator-dependent variability, it is straightforward and widely applicable in clinical practice. Radiomics analysis for DWI and SWI, which may provide more information than ROI measurement, will be explored in our further study.

## Conclusions

In conclusion, lower Ki‑67 index, higher pre‑chemotherapy ITSS grade, and lower post‑chemotherapy ADC parameters were associated with R/R PCNSL. Other pathological indicators, cMRI and pre‑chemotherapy ADC parameters showed limited predictive value for R/R PCNSL. Additionally, the predictive performance of post‑chemotherapy ADC parameters, including rADC_min−post_, rADC_mean−post_, rADC_min−change_, and rADC_mean−change_, was comparable to that of pre-chemotherapy ITSS grade for R/R PCNSL, but superior to that of Ki-67 index. Our study revealed that pre-chemotherapy ITSS grade from SWI and post-chemotherapy ADC parameters from DWI may aid in predicting R/R PCNSL.

## Electronic supplementary material

Below is the link to the electronic supplementary material.

Supplementary Material 1

## Data Availability

The data and materials that support the findings of this study are available from the corresponding author upon reasonable request.

## Abbreviations

PCNSL: Primary central nervous system lymphoma
R/R: Relapsed/refractory
HD-MTX: High-dose methotrexate
cMRI: Conventional magnetic resonance imaging
SWI: Susceptibility-weighted imaging
ITSS: Intra-tumoral susceptibility signal
DWI: Diffusion-weighted imaging
ADC: Apparent diffusion coefficient
DLBCL: Diffuse large B-cell lymphoma
CT: Computed tomography
PET-CT: Positron emission tomography/computed tomography
CR: Complete response
PR: Partial response
PD: Progressive disease
SD: Stable disease
GCB: Germinal center B-cell-like
T1WI: T1-weighted imaging
T2WI: T2-weighted imaging
CE-T1WI: Contrast-enhanced T1-weighted imaging
FLAIR: Fluid-attenuated inversion recovery imaging
PACS: Picture archiving and communication system
ROI: Region of interest
NAWM: Normal-appearing white matter
ROC: Receiver operating characteristic
AUC: Area under the curve
ICC: Intra-class correlation coefficient
