# Supplementary material for: Prediction of relapsed/refractory primary central nervous system lymphoma using pre-chemotherapy ITSS grade on SWI and pre-/post-chemotherapy ADC parameters on DWI
Source: Cancer Imaging. 2026 Apr 30;26:80. doi: 10.1186/s40644-026-01042-8 (PMC13281242; doi:10.1186/s40644-026-01042-8)
Supplement: Supplementary file 1 — Supplementary Material 1 [file 40644_2026_1042_MOESM1_ESM.docx]

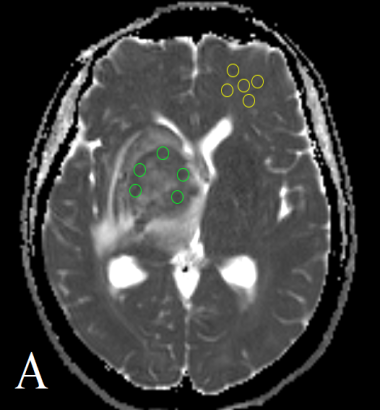

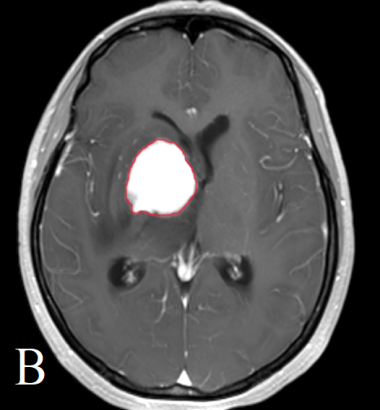

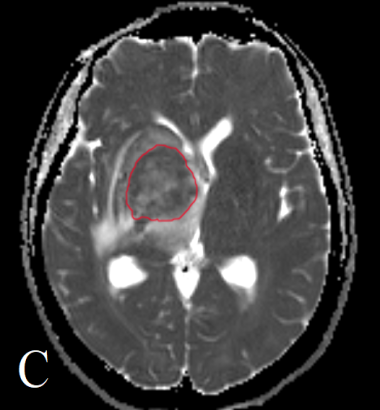


Supplemental Fig. 1. An example of ADC measurements. A Minimum ADC (green) and normal-appearing white matter ADC (yellow). B and C Mean ADC (red).


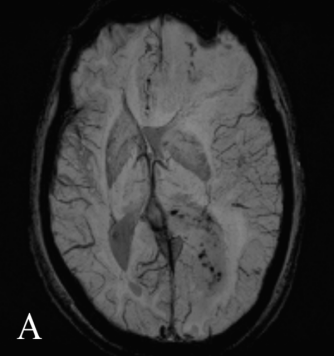

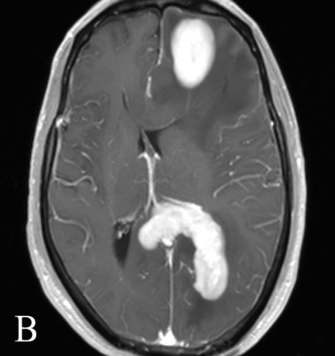

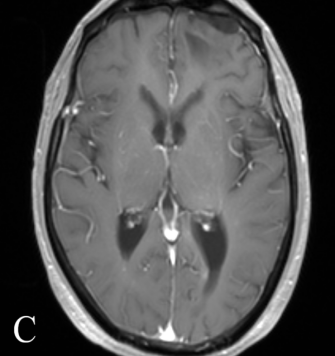

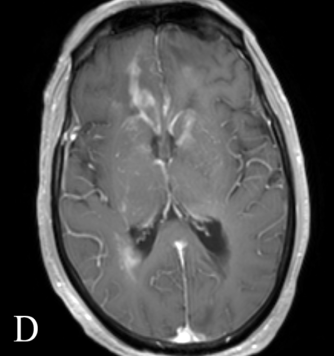


Supplemental Fig. 2. A 67-year-old male with relapsed/refractory PCNSL. SWI showed multiple low-signal-intensity dotlike structures within the lesions, ITSS grade 3 (A). CE-T1WI revealed two prominently homogeneous contrast-enhancing tumors in the left frontal lobe and the splenium of the corpus callosum extending to the left occipital lobe (B). The enhancing lesions almost disappeared after 2 cycles of first-line chemotherapy (C). After completion of the full course of first-line chemotherapy, there were new patchy enhancing lesions in basal ganglia and the peripheral ventricles (D), and the efficacy was evaluated as PD.

Supplemental Table 1. Comparison of cMRI characteristics between the two groups.

|  | R/R group  (n = 40) | Non-R/R group  (n = 37) | *P* value |
| --- | --- | --- | --- |
| Tumor location |  |  |  |
| supratentorial/infratentorial | 33/7 | 33/4 | 0.402 |
| cortical/deep | 18/22 | 13/24 | 0.378 |
| Number |  |  | 0.818 |
| single | 12 | 12 |  |
| multiple | 28 | 25 |  |
| Tumor size |  |  | 0.742 |
| maximum diameter (mean±SD) | 3.72 ± 1.47 | 3.61 ± 1.50 |  |
| Necrosis |  |  | 0.221 |
| Yes | 7 | 3 |  |
| No | 33 | 34 |  |
| Peritumoral edema |  |  | 0.758 |
| mild | 5 | 4 |  |
| moderate | 7 | 9 |  |
| severe | 28 | 24 |  |
| Enhancement pattern |  |  | 0.220 |
| homogeneous | 31 | 24 |  |
| heterogeneous | 9 | 13 |  |
| Enhancement margins |  |  | 0.233 |
| clear | 29 | 31 |  |
| blurred | 11 | 6 |  |

Note: R/R, relapsed and refractory; SD, standard deviations.
